# Neurological Evaluation of Geriatric Patients Being Treated for COVID-19

**DOI:** 10.5152/eurasianjmed.2022.21099

**Published:** 2022-02-01

**Authors:** Ahmet Adiguzel, Unal Ozturk

**Affiliations:** Department of Neurology, University of Health Sciences, Diyarbakir Gazi Yasargil Education and Research Hospital, Diyarbakır, Turkey

**Keywords:** COVID-19, neurological disorders, neurological manifestations

## Abstract

**Objective:** The clinical presentation of coronavirus disease 2019 may be more severe in individuals with diagnoses such as neurodegenerative diseases and cerebrovascular disease, which may occur at an advanced age, among the underlying chronic neurological disorders. In this study, we analyzed the incidence of underlying neurological disorders, the clinical process, the effects on prognosis, duration of hospitalization, and clinical parameters such as mortality and the incidence of neurological manifestations that occurred in the study group after being infected and their relationship with the prognosis in patients hospitalized due to coronavirus disease 2019.

**Materials and Methods**: This is a retrospective and single-centered study. Individuals aged 65 years and older whose diagnosis of coronavirus disease 2019 was confirmed and who were hospitalized for treatment were included in the study.

**Results**: A total of 282 individuals were included in the study. Neurological manifestaitons were observed in 217 (77.0%) patients, and 131 (46.5%) patients had a neurological disorders in their medical history. Of the 58 patients in intensive care, 36 (12.8%) had a positive history of neurological disorders (*P* = .006). The incidences of diseases common in advanced age were 22 (7.8%) for dementia, 37 (13.1%) for cerebrovascular disease, and 4 (1.4%) for movement disorders. The most common symptom were myalgia in 67 (23.8%) patients.

**Conclusion**: The clinical presentation was more severe and the risk of being treated in the intensive care unit was higher in individuals with a history of neurological disorders and neurological manifestations. Furthermore, patients who developed neurological manifestations had a greater risk of mortality and longer periods of hospitalization.

## Main Points

- Coronavirus disease 2019 hospitalization period is longer and the need for intensive care is greater in individuals with underlying neurological diseases.
- Mortality risk was found at the highest rate in individuals with dementia.
- The most common symptom was myalgia. The clinical presentation was severe and the mortality risk was high in patients with confusion and delirium.

## Introduction

Severe acute respiratory syndrome coronavirus-2 (SARS-CoV-2) was first identified in Wuhan, China, in December 2019.^1^ Coronavirus disease 2019 (COVID-19), as it is better known, was declared a pandemic by the World Health Organization (WHO) on March 11, 2020, after infecting nearly 2 500 000 individuals.^2^ The most common clinical symptoms of COVID-19 are fever, cough, dyspnea, and malaise. Acute respiratory distress syndrome presenting with severe pneumonia, extrapulmonary organ damage, and even death may occur in some patients.^3^ The elderly population, especially those with comorbidities such as hypertension, diabetes mellitus (DM), and chronic obstructive pulmonary disease, are more likely to develop complications secondary to the disease.^4^ However, no clear consensus exists on which individuals with neurological diagnoses treated for COVID-19 are more at risk and what their effects on morbidity and mortality are. Considering that most of the clinically worsening and disabling cerebrovascular diseases (CVD) and neurodegenerative diseases, such as dementia and Parkinson’s disease, are more common in individuals aged 65 and older, we can predict that this group will have a clinically poor prognosis depending on age during the pandemic.

The central nervous system (CNS) has been a critical target in some infectious diseases whose clinical manifestations and symptoms occur because of direct transmission or as a secondary consequence of infection. In addition, the peripheral nervous system (PNS) is negatively affected by numerous factors including weakened immunity during the infection, prolonged hospitalization, and decreased mobilization.^5^ Individuals infected by the novel coronavirus may initially develop non-specific neurological manifestations, and if the clinicians do not notice these symptoms, the clinical presentation may become more complicated in the subsequent processes. For example, in the geriatric group, which is more vulnerable to complications associated with COVID-19, simple headaches at the initial stage could be the first manifestation of fatal encephalitis. This study aims to determine the incidence of neurological diagnoses in the medical histories of patients over 65 years of age hospitalized in our center due to COVID-19 as well as the neurological signs and symptoms that occur during hospitalization and to investigate the effects of the outcomes on the prognosis.

## Materials and Methods

This study was approved by the ethics committee of University of Health Science, Diyarbakır Gazi Yaşargil Education and Research Hospital on September 11, 2019, with decision number 545 and Helsinki Declaration principles were followed.

### Study Design and Participants

This retrospective study was planned as a single-center study. Patients aged 65 years and older whose diagnosis of SARS-CoV-2 was confirmed according to WHO guidelines and who were hospitalized between April 1, 2020, and July 1, 2020, in Turkey at a full-fledged tertiary center, which was selected by the Ministry of Health to serve as a pandemic hospital during the pandemic, were included in the study group.^6^ Clinical, laboratory, and imaging results of the patients hospitalized in our center due to SARS-CoV-2 were checked daily by an infectious disease and thoracic disease specialist. Nasopharyngeal and oropharyngeal samples were taken on the first day from inpatients. The diagnosis was made based on clinical symptoms, lung computerized tomography, and nasopharyngeal swabs taken for reverse transcription polymerase chain reaction (RT-PCR) to confirm the presence of SARS-CoV-2.

Neurological examination was performed in patients with neuropathic complaints. Participants with a previous diagnosis of hypokinetic (e.g., Parkinson’s disease) or hyperkinetic (e.g., essential tremor) movement disorders were evaluated under the category of movement disorders. Headache is one of the most common symptoms in the patients followed up. The diagnosis of primary headache was made according to the criteria of The Third edition of the International Classification of Headache Disorders. In general, findings supporting the diagnosis such as paresis, paresthesia starting in the distal extremities, pain, numbness, tingling, burning, pins and needles, throbbing, glove-sock-type hypoesthesia were observed in patients. The diagnosis of delirium was made according to the Diagnostic and Statistical Manual of Mental Disorders, Fifth Edition. According to the third edition of the International Classification of Sleep Disorders, newly developed insomnia was categorized under sleep disorder.

The patients’ existing neurological diagnoses and newly emerging symptoms were observed and confirmed by 2 neurologists. First, the medical histories of the patients were investigated for neurological diagnoses and then the definitive diagnoses of the patients and the appropriateness of the drugs used for those diagnoses were examined in the electronic file system. Thus, their diagnoses were confirmed twice. Neurological manifestations that occurred during hospitalization were investigated and recorded in the electronic file by clinicians who were monitoring the patients daily.

### Statistical Analysis

Statistical analysis was conducted using Statistical Package for the Social Sciences software^®^ for Windows, version 20.0 software (IBM Corp.; Armonk, NY, USA). The chi-square test was used for comparison of categorical variables. Normal distribution of continuous data was determined using the Kolmogorov–Smirnov test. Spearman test was used for nonparametric data, and the Pearson test was used for parametric data. *P* < .05 was considered statistically significant.

## Results

### Demographic and Clinical Features

Of 1680 inpatients with a confirmed diagnosis of SARS-CoV-2, 282 people aged 65 and older were included in the study, including 126 (44.7%) women and 156 (55.3%) men. Mean age of the participants was 73.7 ± 6.8 years, mean age of those with a diagnosis of a neurological disorders in their medical history was 74.1 ± 6.6 years, and mean age of those who developed neurological manifestations was 73.8 ± 6.7 years (*P* = .825). The overall mean hospitalization time of the patients was 10.9 ± 11.3 days, and the mean hospitalization times of those with and without neurological diseases were 12.6 ± 15.0 and 9.5 ± 6.3 days (*P* = .022), respectively. Similarly, the mean hospitalization times of patients with and without neurological manifestations were 12.3 ± 12.5 and 6.1 ± 2.0 days, respectively (*P* = .000).

A total of 58 (20.6%) patients were treated in the intensive care unit (ICU). Based on clinical severity, people with underlying neurological diseases had a greater risk of being hospitalized in the ICU. Accordingly, 58 (20.6%) patients in total were hospitalized in the ICU, including 36 (12.8%) with a known neurological diagnosis and 22 (7.8%) without any diagnosis (*P* = .006). We found that the number of patients who developed neurological manifestations in the ICU was 58 (20.6%), that is, all patients in the ICU had a neurological sign or symptom (*P* = .000). The incidence of mortality in those with and without neurological diagnoses in their medical histories was 30 (10.6%) and 18 (6.4%), respectively (*P *= .011). In all patients with mortality, neurological manifestations were detected in 48 (17.0%), and they were statistically significant (*P* = .000). Other demographic and clinical data of the study group were investigated in detail (Table 1).

### Outcomes of Neurological Diagnoses and Clinical Manifestations

During the study, we found that some patients had multiple neurological diagnoses (such as dementia + CVD) or multiple neurological manifestations (such as headache + hyposmia). We divided patients into 2 groups based on clinical severity as those who were and were not hospitalized in the ICU and then we analyzed the relationship between these 2 groups in terms of neurological history and newly developing neurological manifestations. We investigated the frequency of neurological diagnoses in patients hospitalized in the ICU, namely those with a severe clinical presentation, and its relationship with the other group. Dementia was present in the medical history of 10 (3.5%) (*P* = .005) patients, movement disorders (MD) in 3 (1.1%) (*P* = .028), CVD in 15 (5.3%) (*P* = .002) [ischemic 14 (5.0%) (*P* = .002) and hemorrhagic 1 (0.4%) (*P* = .604)], headache in 5 (*P* = .256), polyneuropathy (PNP) in 10 (3.5%) (*P* = .176), sleep disorders in 2 (0.7%) (*P* = .359), and epilepsy in 1 (0.4%) (*P* = .604) patient’s medical history.

We investigated the frequency of the signs and symptoms that occurred and were detected during hospitalization in patients in the ICU. Accordingly, headache was found in 10 (3.5%), vertigo-dizziness in 10 (3.5%), hyposmia-anosmia in 8 (2.8%), neuropathic complaints in 15 (5.3%), sleep disturbance in 15 (5.3%), change of consciousness in 27 (9.6%), delirium in 20 (7.1%), myalgia in 17 (6.0%), and other neurological findings were detected in 1 (0.4%) patients.

Considering the relationship between mortality and medical history in all patients, we see that 10 of the 22 patients with dementia died (*P* = .001). We examined the relationship of other neurological disorders and manifestations with clinical severity and mortality in detail (Table 2).

Considering the relationship between mortality and manifestations, mortality rates of patients who developed confusion and delirium were calculated to be 22/30 (73.3%) and 15/31 (48.4%), respectively, and these 2 symptoms pose a significant risk for mortality (*P* = .000).

## Discussion

### General Structure and Neuroinvasion Mechanisms of SARS-CoV-2

Coronaviruses have an average diameter of 100 nm; they are named after their characteristic crown-shaped microscopic appearance and are positive-sense single-stranded RNA viruses.^4^ Betacoronaviruses, such as SARS-CoV, Middle East Respiratory Syndrome-Coronavirus (MERS-CoV), and SARS-CoV-2, are the most pathogenic species for humans in this virus family of 4 subfamilies. In fact, SARS-CoV and SARS-CoV-2 have similar viral properties, for example, both target the same angiotensin-converting enzyme 2 receptor, which leads to predominantly similar pulmonary symptoms caused by both viruses.^7^ Angiotensin-converting enzyme 2 has a membrane-bound protein structure, which is expressed in different organs, including skeletal muscle and brain.^8^ Coronavirus particularly affects the respiratory system in humans, but due to its ability of neuroinvasion, it can spread to the CNS. In vivo and in vitro studies revealed the neurotropism of human coronaviruses; some viruses are invasive as they target oligodendrocytic and neuroglial cell lines and thus they are present in CNS.^9^ Another possible pathway is to access the CNS by retrograde transport from infected peripheral neurons.^10^

### Patients with a Neurological Diagnosis and COVID-19

At present, stating and proving the frequency of neurological manifestations of SARS-CoV-2 as well as which findings and symptoms have what kind of long- or short-term effects on the prognosis is difficult as the pandemic is still ongoing. A new study is published on this topic almost every day. Therefore, clearer and more comprehensive data will be obtained in the coming years. However, in the present study, we aimed to address the association between neurology and COVID-19 in a different aspect, which has not yet been sufficiently addressed in literature. Considering this from a different perspective, we analyzed our patients wondering what the prognosis would be if individuals with a diagnosis of a neurological disease and using neurological drugs are infected with the novel coronavirus, which underlying neurological diagnosis would stand out in these patients, and to what extent it would affect the process. Furthermore, we evaluated the neurological manifestations that occurred in patients during the treatment period. Since the majority of serious neurological diseases are observed in the geriatric age group, we formed our study group with patients aged 65 years and older. Some patients with COVID-19 may also develop nonspecific or other more serious and specific neurological manifestations, such as headache and vertigo at any stage of infection.

In a single-centered study conducted in China, neurological findings and symptoms of 214 patients hospitalized due to COVID-19 were investigated. Patients were divided into 2 groups based on 50 years of age and 124 (57.9%) individuals were 50 years old and above. In that study, the numbers of those with and without a severe clinical presentation were 64 (72.7%) and 60 (47.69%), respectively, in those aged ≥50 and 24 (27.3%) and 66 (52.69%), respectively, in those aged <50 (*P* < .001).^11^ This result shows that the frequency of severely ill patients increases with age. In addition, as a result of this study, it has been reported that the risk of CVD and sudden change in consciousness is higher in patients with severe infection.^12^

To assess individuals with an underlying neurological disease, which is the main aim of this study, we included patients hospitalized in the ICU in the category of severely ill patients. When we consider the relationship between patients with a neurological diagnosis and patients hospitalized in the ICU, the number of patients with dementia was 10/58 (17.2%) (*P* = .005) and with CVD was 15/58 (25.9%) (*P* = .002). This result suggests that the clinical presentation would be severe if individuals with dementia and CVD, the leading causes of major disability in geriatric patients, are infected with COVID-19. We can say that it causes the immune system to be weakened and consequently become more defenseless against infectious diseases along with adverse factors that we encounter more often in this group of patients, such as predisposition to aspiration, malnutrition, and vitamin deficiencies due to impaired oral intake, as well as lack of hygiene and self-care. Referring to the mortality index of these 2 diseases, 10/22 (45.4%) (*P* = .001) were individuals with dementia and 10/37 (27%) were individuals with CVD (*P* = .071). Fewer individuals, 4 in total, had a diagnosis of MD compared to dementia and CVD. Mortality and hospitalization in the ICU rates of this group were 2/4 (50%) (*P* = .136) and 3/4 (75%) (*P* = .028), respectively. Based on these results, patients with dementia particularly had a greater risk of both mortality and hospitalization in the ICU. A similar result was emphasized in a study conducted in China that concluded that elderly individuals are more susceptible to severe illnesses and that the risk of hospitalization in the ICU and mortality is higher in these individuals.^11^ In a literature review, 4021 cases of COVID-19 were investigated, where 1052 (26.2%) individuals were aged 60 years or older; the mortality index for individuals <60 and ≥60 years of age was reported to be 1.4% and 5.3%, respectively.^13^

Animal experiments have shown that SARS-CoV reached the brain through the olfactory pathway and initiated neurodegeneration.^14^ This result could be a signal of a danger that we may experience in the coming years. This is because considering that millions of young and middle-aged individuals are infected in this period, the incidence of neurodegenerative diseases in infected individuals or exposure to neurodegenerative diseases at a younger age will increase. From the reverse perspective, if such a bad scenario occurs in humans, the incidence of neurodegenerative diseases in individuals who previously had SARS-CoV-2 infection could increase and the age of onset of the diseases could decrease; moreover, their clinical presentation may be more severe and progressive.

Individually considering headaches (tension type, migraine, etc.), epilepsy, PNP, sleep disorders, among other underlying neurological diseases in this study group, no significant relationship was detected between these diseases and mortality index and hospitalization in the ICU. The neurological presentation of these diseases had a milder course than the 3 diseases above and better response to treatment may have had a role in obtaining such a result. An interesting result was also found regarding PNP here because PNP usually occurs secondary to another disease (malignancy and DM). Therefore, we can consider that the clinical prognosis of these patients may be worse and more open to complications in combination with COVID-19. However, to verify the validity of this hypothesis, the number of patients should be increased and the time to PNP diagnosis should be investigated.

### Neurological Findings and COVID-19

Neurological manifestations have a very wide range and can occur at any stage of the clinical presentation of coronavirus. In addition to mild manifestations, such as headache and vertigo, coronaviruses have also been shown to induce various neurological diseases with serious consequences, such as PNP, encephalitis, and ischemic stroke.^15^ In a study that specifically investigated the neurological manifestations of COVID-19, neurological manifestations were divided into 2 groups: those associated with CNS and those associated with PNS. The numbers of patients with CNS symptoms were 53 (24.8%), 27 (30.7%), and 26 (20.6%) (*P* = .094) in the entire group of patients, severely ill patients, and not severely ill patients, respectively, while for PNS symptoms, these numbers were 19 (8.9%), 7 (8.0%), and 12 (9.5%), respectively (*P* = .691), and the numbers of those without any neurological manifestations were 78 (36.4%), 40 (45.5%), and 38 (30.2%), respectively (*P* < .05).^12^ These results show that neurological manifestations are less common in patients who are not severely ill.

Similar results were found in 2 different studies conducted 15 and 17 years ago, one of the first case series on this topic. The RNA of the virus was detected in the cerebrospinal fluid of a patient with SARS, while brain tissue samples taken from 8 patients with SARS for autopsy revealed the presence of SARS-CoV with immunohistochemistry, electron microscopy, and RT-PCR.^16^

A study published on olfactory disorders in 2006 has been reported that post-viral anosmia was one of the leading causes of loss of sense of smell in adults, and its incidence has increased up to 40%.^17^ Olfactory nerve endings and olfactory bulb located in the nasal cavity are thought to serve as a connecting channel between the CNS and nasal cavity.^18^ This last scenario serves as a basis for the anosmia and hyposmia findings in many patients with COVID-19.^18^ In an experimental study, which supports this theory, the removal of the olfactory bulb in mice resulted in coronavirus’ partial invasion of CNS.^19^ The underlying cause is primarily the nasal congestion and damage to olfactory cells caused by mucosal congestion.^17^ Similar information was known before the pandemic, but it did not attract significant attention or was overlooked as few cases had been encountered. However, during this period, we have seen that the novel coronavirus causes impairment in the senses of taste and smell without the mechanisms mentioned above, in other words, without causing rhinorrhea or nasal congestion.^4^ In another study that supports this remarkable data, in 18.2% of the participants who did not have rhinorrhea or nasal obstruction, olfactory dysfunction was found in as high as 79.7% of them.^20^

In our study group, the numbers of individuals with hyposmia–anosmia and hypogeusia were found to be 27 (9.6%) and 24 (8.5%), respectively. Considering our data, in 4 out of 27 patients with hyposmia, an olfactory disorder preceded pulmonary complaints, suggesting that the neuroinvasion process could develop in a very short time. We found that this important finding is supported by another study. A total of 417 COVID-19 patients with mild and moderate clinical presentations were included in a multi-centered study conducted in Europe. The incidence of smell and taste disorders was found to be very high, 85.6% and 88.8%, respectively. Of those with smell disorders, 79.6% were anosmic and 20.4% were hyposmic. Moreover, olfactory dysfunction preceded other symptoms in 11.8% of the individuals in this group.^20^

If we consider the symptoms and findings of our patients, headache was found in 59 (20.9%), vertigo in 36 (12.8%), and myalgia in 67 (23.8%) individuals. These 3 findings were independent of clinical severity. Thus, their rates between the groups that were and were not hospitalized in the ICU were analyzed and found to be close to each other. The fact that these findings are common in almost all viral infections and in all age groups could explain the lack of correlation with clinical severity.

In a study where neurological signs and symptoms were divided into 2 main categories, the most common CNS-linked symptoms in order of frequency were dizziness in 36 (16.8%), headache in 28 (13.1%), and consciousness impairment in 16 (7.5%) patients. Among the PNS-linked symptoms, the most common ones were hypogeusia in 12 (5.6%), hyposmia in 11 (5.1%), and neuralgia in 5 (2.3%) patients. In addition, myalgia was observed in 23 (10.7%) patients in this group.^12^

The numbers of patients with sleep disturbance and confusion were 46 (16.3%) and 30 (10.6%), respectively, and the numbers and rates of hospitalization in the ICU in patients with sleep disturbance and confusion were 15/46 (32.6%; *P* = .026) and 27/30 (90.0%; *P* = .000), respectively. These results show that the incidence of these 2 symptoms was greater in patients hospitalized in the ICU. Delirium was another symptom that we observed more often in individuals hospitalized in the ICU. The proportion of patients in the ICU who developed delirium was 20/31 (64.5%) (*P* = .000).

We might actually say that it is an expected finding, because delirium is usually one of the undesirable findings that is very common in the ICU. Since advanced age is a well-defined independent risk factor for delirium, those who are at the greatest risk for severe pulmonary disease associated with COVID-19 are also at the greatest risk for delirium.^21^ Therefore, since our group comprised geriatric and critically ill patients, we obtained results in line with literature. According to the data on this topic, delirium can be observed at rates ranging from 50% to 70% in critically ill patients and from 10% to 15% in all groups of hospitalized patients.^22^

In another study published in April 2020, the incidence of neurological manifestations in the group of patients with severe clinical presentation was reported as follows: confusion in 26 (65%), agitation in 40 (69%), dysexecutive syndrome in 14 (36%), abnormal corticospinal tract signs in 39 (67%), and ischemic stroke in 3 (23%) patients.^23^

In patients with COVID-19, delirium is believed to occur due to multiple reasons, including direct viral invasion of the CNS, the secondary effect of insufficiency of other organ systems with induction of inflammatory mediators, sedative agents, prolonged periods of mechanical ventilation, and environmental factors such as social isolation.^21^

If we consider the overall results of this study, the duration of hospitalization was longer for individuals with a previously known diagnosis of a neurological disease or the group that developed neurological manifestations in the hospital, and individuals in this group had a higher risk of becoming severely ill.

Although the study population is large, it includes patients admitted to a single hospital and does not include demographically different populations across the country.

In conclusions, in this study conducted with patients hospitalized due to COVID-19, we see that the hospitalization period is longer and the need for intensive care is greater in individuals with underlying neurological diseases. Mortality risk was found at the highest rate in individuals with dementia. Clinical presentation is more severe in patients with dementia and CVD. The most common symptom was myalgia, and the clinical presentation was severe and the mortality risk was high in patients with confusion and delirium. We tried to shed light on the consequences of the association between the clinical presentations of COVID-19 and neurological manifestations through this study conducted during the pandemic, which is still ongoing worldwide.

## Figures and Tables

**Table 1.** Characteristics of Patients with Neurological History and Neurological Findings

	Neurological History+	Neurological History−	*P*	Neurological Symptom+	Neurological Symptom−	*P*	Total
Mean age	74.1 ± 6.6	73.4 ± 6.9	.338	73.8 ± 6.7	73.6 ± 7.0	.825	73.7 ± 6.8
ICU+, n (%)	36 (12.8%)	22 (7.8%)	.006	58 (20.6%)	0 (0%)	.000	58 (20.6%)
Mean length of hospitalization	12.6 ± 15.0	9.5 ± 6.3	.022	12.3 ± 12.5	6.1 ± 2.0	.000	10.9 ± 11.3
Mortality, n (%)	30 (10.6%)	18 (6.4%)	.011	48 (17.0%)	0 (0%)	.000	48 (17.0%)
Gender, F/M	58 (20.6%)/73 (25.9%)	68 (24.1%)/83 (29.4%)	.497	96 (34.0%)/121(42.9%)	30(10.6%)/35(12.4%)	.447	126(44.7%)/156 (55.3%)
Neurological findings	115 (40.8%)	102 (36.2%)	.000	-	-	-	217 (77.0%)
Neurological history	-	-	-	115 (40.8%)	16 (5.7%)	.000	131 (46.5%)

M, male; F, female.

**Table 2.** Relationship of Diagnosis and Symptoms with Clinical Parameters

Neurological Diagnosis	ICU+ (n = 58)	ICU– (n = 224)	*P*	Expired (n = 48)	Survived (n = 234)	*P*	Total (n = 282)
Dementia	10 (3.5%)	12 (4.3%)	.005	10 (3.5%)	12 (4.3%)	.001	22 (7.8%)
MD	3 (1.1%)	1 (0.4%)	.028	2 (0.7%)	2 (0.7%)	.136	4 (1.4%)
CVD	15 (5.3%)	22 (7.8%)	.002	10 (3.5%)	27 (9.6%)	.071	37 (13.1%)
Ischemic	14 (5.0%)	19 (6.7%)	.002	9 (3.2%)	24 (8.5%)	.082	33 (11.7%)
Hemorrhagic	1 (0.4%)	3 (1.1%)	.604	1 (0.4%)	3 (1.1%)	.528	4 (1.4%)
Headaches	5 (1.8%)	29 (10.3%)	.256	5 (1.8%)	29 (10.3%)	.461	34 (12.1%)
Tension type	5 (1.8%)	22 (7.8%)	.506	5 (1.8%)	22 (7.8%)	.501	27 (9.6%)
Migraine	0 (0%)	5 (1.8%)	.313	0 (0%)	5 (1.8%)	.391	5 (1.8%)
Others	0 (0%)	2 (0.7%)	.630	0 (0%)	2 (0.7%)	.688	2 (0.7%)
PNP	10 (3.5%)	26 (9.2%)	.176	10 (3.5%)	26 (9.2%)	.060	36 (12.8%)
Sleep disorders	2 (0.7%)	4 (1.4%)	.359	0 (0%)	6 (2.1%)	.323	6 (2.1%)
Epilepsy	1 (0.4%)	3 (1.1%)	.604	1 (0.4%)	3 (1.1%)	.528	4 (1.4%)
**Symptoms**							
Headache	10 (3.5%)	49 (17.4%)	.282	7 (2.5%)	52 (18.4%)	.161	59 (20.9%)
Dizziness/vertigo	10 (3.5%)	26 (9.2%)	.176	5 (1.8%)	31 (11.0%)	.397	36(12.8%)
Hyposmia/anosmia	8 (2.8%)	19 (6.7%)	.164	7 (2.5%)	20 (7.1%)	.152	27 (9.6%)
Hypogeusia	0 (0%)	24 (8.5%)	.003	0 (0%)	24 (8.5%)	.009	24 (8.5%)
Neuropathic complaints	15 (5.3%)	42 (14.9%)	.154	15 (5.3%)	42 (14.9%)	.033	57 (20.2%)
Sleep disorders	15 (5.3%)	31 (11.0%)	.026	10 (3.5%)	36 (12.8%)	.232	46 (16.3%)
Confusion	27 (9.6%)	3 (1.1%)	.000	22 (7.8%)	8 (2.8%)	.000	30 (10.6%)
Delirium	20 (7.1%)	11 (3.9%)	.000	15 (5.3%)	16 (5.7%)	.000	31(11.0%)
Myalgia	17 (6.0%)	50 (17.7%)	.173	17 (6.0%)	50 (17.7%)	.032	67 (23.8%)
Others	1 (0.4%)	1(0.4%)	.370	1 (0.4%)	1 (0.4%)	.312	2 (0.7%)

ICU, intensive care unit; MD, movement disorders; CVD, cerebrovascular disease; PNP, polyneuropathy.
